# Genome-Wide Expression Analysis of a Spinal Muscular Atrophy Model: Towards Discovery of New Drug Targets

**DOI:** 10.1371/journal.pone.0001404

**Published:** 2008-01-02

**Authors:** Sheena Lee, Arzu Sayin, Stuart Grice, Howard Burdett, Dilair Baban, Marcel van den Heuvel

**Affiliations:** 1 Medical Research Council (MRC) Functional Genetics Unit, Department of Physiology, Human Anatomy and Genetics, University of Oxford, Oxford, United Kingdom; 2 OXION Initiative, Oxford Centre for Gene Function, Department of Human Anatomy and Genetics, University of Oxford, Oxford, United Kingdom; University of Giessen, Germany

## Abstract

Spinal Muscular Atrophy is a recessive genetic disease and affects lower motor neurones and muscle tissue. A single gene is disrupted in SMA: SMN1 activity is abolished but a second copy of the gene (SMN2) provides limited activity. While the SMN protein has been shown to function in the assembly of RNA-protein complexes, it is unclear how the overall reduction in SMN activity specifically results in the neuromuscular phenotypes. Similar to humans, reduced *smn* activity in the fly causes earliest phenotypes in neuromuscular tissues. To uncover the effects of reduced SMN activity, we have studied gene expression in control and diseased fly tissues using whole genome micro-arrays. A number of gene expression changes are recovered and independently validated. Identified genes show trends in their predicted function: several are consistent with the function of SMN, in addition some uncover novel pathways. This and subsequent genetic analysis in the fly indicates some of the identified genes could be taken for further studies as potential drug targets for SMA and other neuromuscular disorders.

## Introduction

Whole genome expression analysis of animals and tissues offers a novel way of penetrating functional changes in diseased versus normal tissues. Such analysis will map direct and indirect effects of the disease state on gene expression and could identify novel gene targets for drug interference.

Genome wide expression arrays have been made possible by the annotation and gene predictions of whole genome sequence data for human and a large number of model organisms. The genome sequencing work and subsequent species comparisons have also indicated that the genetic differences between species are smaller than anticipated. Both the total number of genes identified and, interestingly, the variety of encoded proteins (and domains) are smaller than expected. Combining these findings with functional genetic analysis has led to the conclusion that for most tissues a group of highly conserved genes is required for differentiation and subsequent function. These genes are expressed and functional in the investigated tissues across the evolutionary tree of animal species. Tissue specific analysis can thus be performed in analogous tissues using a wide variety of model organisms, concentrating on these highly conserved genes.

We have decided to probe the function of known human disease genes by creating and studying mutants in homologues of such genes, in simple model organisms. Here we present a study of gene expression in a simple model organism to identify novel gene pathways that are differentially regulated in normal versus mutant, i.e. diseased, states. The identification of such pathways could potentially lead to the identification of novel drug targets.

SMA is a common recessive genetic disease affecting 1:8000-10.000 across the human population and is characterized by a specific loss of lower motor neurones and muscle atrophy. The genetic cause was identified as the Survival Motor Neurone gene (SMN), however, in the disease situation, SMN protein activity is never completely lost, instead is reduced [1]. The human genome contains two copies of the SMN gene, the activity of the first copy (SMN1) is genetically affected in patients and they therefore rely totally on the activity contributed by the second copy (SMN2). However SMN2 produces less functional protein to varying degrees. The level of remaining SMN activity has been directly correlated to the severity of the phenotypes [2]. In the patient cohort this has lead to distinct groupings, type I being strong, affecting newborns and babies, type II, affecting childhood and type III, affecting late childhood and adults; each group defined on the basis of concrete motor abilities. The generation of an amenable model reflecting human conditions has been complex. The mouse has a single SMN gene and loss of the gene has been shown to lead to early embryonic lethality [3]. Molecular and functional data relating SMN function to SMA have been slow to accumulate and despite the knowledge of the genetic basis of the disease, little is known about the neuromuscular background of the phenotypes.

The SMN gene is expressed ubiquitously in all animals investigated [4]. Extensive biochemical analysis concluded that it is involved in the assembly of large RNA-protein complexes, one of which is the spliceosome (reviewed in [5], [6]). Although this work was critical in understanding the function of the SMN protein, it doesn't explain the neuronal and/or muscle specificity of SMA. Thus it remains unclear why this protein seemingly required for basal functions in the cell, leads to such specific phenotypes when activity falls below a certain threshold. It is possible that a certain basal function reaches a critical level in the neuromuscular tissues, or SMN might perform a highly specific function in these tissues.

We have generated a fly model for SMA by knocking out the endogenous *smn* gene. Unlike in mice, the knock-out animals are born; the embryos survive on the basis of maternal contribution of transcripts coding for normal Smn protein. Maternal contribution, in the homozygous mutant background, runs out during larval stages and leads primarily to neuromuscular phenotypes before the animals die. Because the only genetic aberration in these animals is the reduction of SMN activity, the observed phenotypes must be due to SMN activity reaching a threshold for neuromuscular functionality. Further characterisation showed that these larvae develop clear external motor problems and cell and physiological defects at the motor neuron-muscle junction [7].

To further the molecular understanding of SMA we used our fly model in a genome wide gene expression analysis. We performed micro array analysis of whole animals and SMA affected tissues across normal and *smn* mutant animals. We used Drosophila whole genome arrays and have produced statistically relevant results. We have validated these using standard methods. In addition we find that, using genetic testing in the fly to investigate functional interactions, novel loci influencing the *smn* mutant phenotypes can be identified using these data, thus opening the path for novel drug target studies.

## Results

### Experimental background

We set out to analyse gene expression in whole animals as well as in individual tissues. The latter potentially poses a problem due to the low quantities of starting material that can be obtained from Drosophila. In order to assess gene expression using micro arrays based on the high density GeneChip system, we needed to establish if it was possible to obtain reliable expression data using small quantities of Drosophila RNA.

We compared the same RNA sample at different dilutions, using the “low starting material” amplification procedures to obtain labelled cRNA for the low concentrations (see Material and methods). The overall yield of labelled RNA and the quality of the hybridisation data are presented in Supplemental Figure S1. Using the Affymetrix two-cycle amplification method we are able to produce ≥15 ug cRNA using starting amounts as low as 0.1 ng; below this level the output drops significantly. The product size decreases as smaller amounts of RNA are used, resulting in 5′ truncation of the cRNA and the subsequent loss in detection of genes with probes directed towards the 5′ end of the gene. On the basis of these tests, we used between 10 ng to 100 ng of starting material in our experiments to minimise 5′ truncation of the cRNA transcripts.

### Experimental set up

Our aim was to compare gene expression changes between normal and *smn* mutant animals and tissues. The strong genetics of Drosophila make it possible to use a “normal” background that is genetically very close to the mutant, this genetic system is thus highly appropriate for genomic analysis. In this study we used a mutant allele of *smn* that was recombined onto an isogenised third chromosome background (see Materials and methods); the *smn* mutant animals or isolated tissues will be homozygous for this allele. The control “normal” background was the same isogenised chromosome in homozygous state, but without the *smn* mutation. The other chromosomes are free to drift genetically in both normal and *smn* mutant strains.

**Figure 1:**
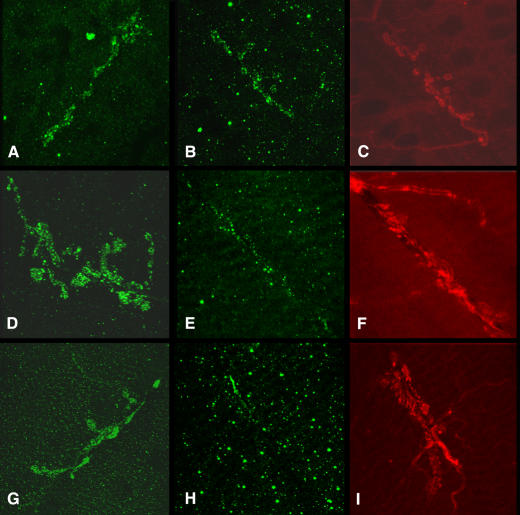
Confocal Scanning Fluorescent images of Drosophila neuromuscular junctions. A to C, early second instar larvae; D to F, late second instar larvae and G to I, third instar larvae (mid stage). A, D and G, normal (+/+) larvae, B, C, E, F, H and I, *smn* mutant larvae (*smn*/*smn*). A, B, D, E, G and H stained for Glutamate receptor subunit; C, F and I stained for a pre-synaptic marker (syntaxin). Comparing the accumulation of receptor clusters in the normal tissue across time to the mutant tissues, it is clear that the mutant displays a reduced number of glutamate receptor clusters across time.

To obtain information on the time frame of molecular changes in the *smn* mutant background and thus when to isolate our samples, we have assessed the neuromuscular tissues in second and third instar larval stages. Motility defects in the *smn* mutant animals are first seen halfway between these stages and are statistically significant by third instar. At this life stage, we have previously reported that clustering of the neurotransmitter receptor is disrupted at the neuromuscular junction (NMJ) [7]. Disruption of neurotransmitter receptor clustering has also been reported in patient tissue [8] and in a recently described mouse model [9]. On our time course, in early second instar larvae the accumulation of clusters of the neurotransmitter receptor, Glutamate receptor IIA (GluR IIA), is similar in mutant animals compared to controls (Figure 1). By late second stage the number and size of clusters in the mutant tissues changes compared to the controls. By the time that the larvae display motility problems (third instar) this difference is exacerbated, leaving very few clusters in the mutant situation. These results argue that the loss of motility in our animals is concomitant with or proceeded by a loss of neurotransmitter receptor clustering at the NMJ after an initially normal pattern. Thus molecular defects are visible from late second instar and increase into third instar stage, note however that even at mid-to-late third instar stage little tissue degeneration is observed (unpublished and see [7]. We used animals at the second and third instar stage for whole animal gene expression analysis and concentrated on individual tissues at the later stage only. The loss in neurotransmitter receptor clustering is most clearly seen at the later stage and since this defect has been shown in other model systems and patients, it would be most interesting to explore in our experimental analysis.

For tissue samples, we dissected muscle tissue and motor neuron cell bodies from mobility impaired third instar larvae. The ventral abdominal muscles were obtained by filleting larvae and removing gut, neurons and dorsal muscles, the ventral muscles were dissected from the remaining cuticle. The cell bodies of the motor neurons are found in the ventral ganglion as part of the brain structure in the larvae; this tissue was dissected out. The whole larvae and tissue samples were processed to generate labelled cRNA.

We used the GC-RMA (Robust Multi-array Analysis) method to normalize the hybridisation results; this method uses the GC content of the probes to reduce variance in the mismatch (control) probe levels as spotted on the Affymetrix arrays [10].

### Hybridisation results

We combined the results across the various samples to identify overlapping changes in gene expression. Any overlap patterns would allow trends to be uncovered. Common disruptions might point to an affected function in SMN deprived tissues. We used a 1.5 fold change in expression as cut-off in these comparisons and stringent statistics. We compared the expression changes for brain and muscle tissues to the whole animal data. If the role of SMN in SMA is linked to a general function that SMN performs in all cells then a transcriptional response to reduced activity might be seen in all cells of the animal. Conversely, changes specific to either muscle and/or brain might point to genes that influence the tissue specificity of SMA (see Gene Pathway Changes paragraph). The number of genes that change in these comparisons is shown in Table 1, and the genes listed in supplemental Figure S2, S3, S4, S5, S6, S7, S8, S9 and S10.

**Table 1:** Table showing the number of gene expression changes at cut-off of ≥1.5 fold, in common between tissues indicated and larvae of different stages.

**Table 1a**	**Larvae/brain comparisons**			
**2^nd^ instar larvae**	**3^rd^ instar larvae**		**Brain**	**No. genes**
No change	No change		Up	436
Up	No change		Up	27
No change	Up		Up	55
Up	Up		Up	22
No change	No change		Down	334
Down	No change		Down	22
No change	Down		Down	13
Down	Down		Down	17
**Table 1b**	**Larvae/Muscle comparisons**			
**2^nd^ instar larvae**	**3^rd^ instar larvae**		**Muscle**	**No. genes**
No change	No change		Up	1184
Up	No change		Up	30
No change	Up		Up	98
Up	Up		Up	28
No change	No change		Down	1363
Down	No change		Down	63
No change	Down		Down	45
Down	Down		Down	30
**Table 1c**		**Brain/muscle comparisons**		
**Brain**		**Muscle**		**No. genes**
Up		Up		217
Down		Down		185

Table showing the number of gene expression changes at cut-off of ≥1.5 fold, in common between tissues indicated and larvae of different stages. Both larval stages, and gene expression changes at ≥1.5 fold that are not seen in larvae are shown, as well as changes in common only between Brain and Muscle samples. All changes have a p-value of ≤0.05 in a two-tailed two-sample Welch t-test.

The number of genes in common between the tissue and whole animal samples is small; each tissue sample itself shows consistently the largest number of gene expression changes (“No change in larvae” row). This might reflect the fact that the lowered levels of *smn* activity specifically affect the neuromuscular tissues; gene expression changes specific to these tissues could fall below the cut-off levels in the whole animal samples. We have previously assessed the sensitivity of the muscle and neuronal tissues in a genetic experiment. A transgene encoding Smn was expressed in a homozygous *smn* mutant background. As expected rescue was almost 100% by ubiquitous expression of the transgene, however, interestingly, significant rescue was also observed when the transgene was expressed solely in muscle and neurons. These results showed clearly that these specific tissues in the fly are more sensitive to the levels of *smn* compared to the rest of the animal [7].

Although it is difficult to concentrate on individual genes at this point, we will highlight some here. Across all samples, three genes encoding cytochrome P450 domains are up-regulated as well as two Glutathione S transferases. Interestingly two genes that encode G-protein-coupled-receptor-like proteins, amenable drug targets, with unknown function are uniformly up-regulated. Amongst the genes that are down regulated, different family members of Glutathione S transferase (CG6776) and cytochrome P450 (or electron transport, CG4511) encoding genes are found. Within the down-regulated genes in the muscle a gene that has predicted RNA cap binding function is found (CG8023).

The larval samples were taken at two time points and this enables us to investigate the changes in gene expression across time. Many genes that have been annotated but not functionally analysed are identified in this way. Within these, a trend is recognizable towards a down-regulation of metabolic and transporter activities. Some genes associated with immune response are found up-regulated. In both brain and muscle samples (and whole larvae) a gene annotated as N-methyl-D-aspartate receptor associated protein is up-regulated, only at the later stage. This might indicate a link with the reduced accumulation of Glutamate Receptors at the mutant NMJs in the mutant animals. A protein known to function in transcript transport and translation control, *spire*, is up-regulated at the earlier stage in muscle only.

When looking only at the tissues samples, it appears that there are more genes showing expression changes in muscle than in brain samples. The overlap between muscle and brain samples will uncover gene expression changes specific to the neuromuscular system. The lower rows in Table 1 show the number of genes changing and the genes are listed in supplemental Figure S2, S3, S4, S5, S6, S7, S8, S9 and S10. Within the down-regulated genes two syntaxin encoding genes are identified. Interestingly, three genes encoding small Heat Shock Proteins are down-regulated especially in the muscle sample.

**Figure 2:**
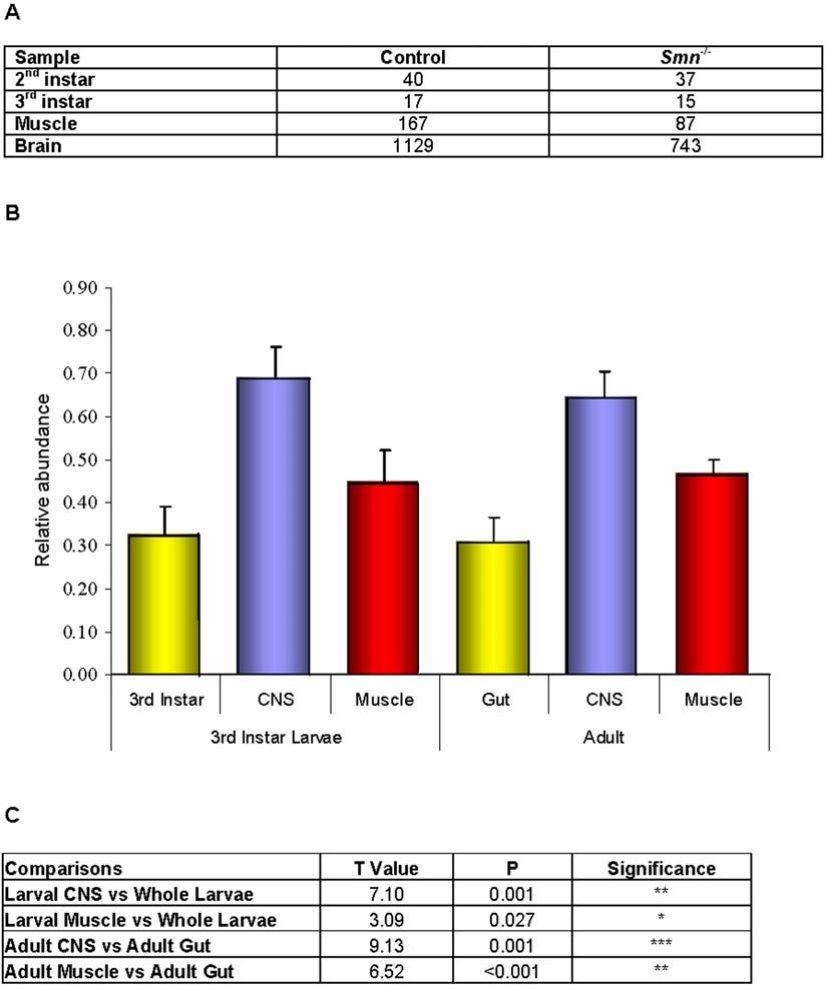
*Smn* gene expression analysis using two different methods. A) Results of micro-array hybridisations. “Sample” column lists the different RNA samples used for cRNA preparation. The control and *smn* mutant relative concentrations are shown. Expression is low in the RNA samples derived from whole animals but is significantly higher in neuromuscular tissues, especially in brain. The expression is reduced by 1.9 and 1.5 fold in the mutant animals in muscle and brain respectively. Note that these tissues are mutant for the somatic gene (a point mutation so transcripts will be produced) while still containing detectable maternal RNA (data not shown). B) Real Time-PCR analysis of *smn* expression across different tissues. The Bar figure shows the relative abundance of *smn* transcript in tissues versus either whole animals (thus containing the isolated tissues; all third instar larval stage derived) or versus isolated gut tissue (all adult derived), all from *smn +/+*. In both Central Nervous System (CNS) and muscle the expression of *smn* is higher. C) The significance of this finding increases in adults

To further understand whether SMN is required specifically in the neuromuscular tissues, we have assessed the expression of the smn gene in these tissues versus the rest of the animal using both our array data and Real-Time Polymerase Chain Reaction (RT-PCR) analysis. This latter method compared the concentration of the smn transcript in larval and adult tissues against non-changing control genes. This analysis clearly shows that expression of smn is higher in larval and adult neuromuscular tissues, with especially high expresssion in the nervous system (Figure 2).

The molecular analysis of the NMJs in the *smn* mutant clearly showed a deregulation of GluR protein accumulation (GluR IIA subunits). Using our data, we assessed the changes in expression of some of the GluR genes (Table 2). GluR IIA and IIB, for which protein is found at the NMJ, are mildly down regulated in *smn* mutant muscle. GluR III (also knows as IIC) is up-regulated in *smn* mutant muscle and no change is seen for GluR IID. All have a p value ≤0.05 of change between controls and *smn* mutant muscle in a Welch t-test.

**Table 2:** Fold changes of GluR genes in *smn* mutant muscle compared to wild type muscle (p value ≤0.05 of a difference between the 2 conditions).

Glutamate receptor	Affymetrix probe ID	Fold change
IIA	143498_at	−2.35
IIB	143926_at	−1.82
III (IIC)	152969_at	4.3
IID	150272_at	no change

Fold changes of GluR genes in *smn* mutant muscle compared to wild type muscle (p value ≤0.05 of a difference between the 2 conditions). The GluR IID gene is also known as KaiRIA.

It has been suggested that the formation of functional receptors requires at least GluR III and one of either IIA or IIB (and possibly other sub units) [11]. The significant up-regulation we observe for GluR III might be a response of the animal to the reduction of receptor activity at the NMJ. The reduction in transcripts for the IIA subunit cannot explain the degree of loss of clustering we see at the neuromuscular junction, arguing the problem in the SMN depleted tissue is not primarily based on transcript reduction.

### Validation of array results

**Figure 3:**
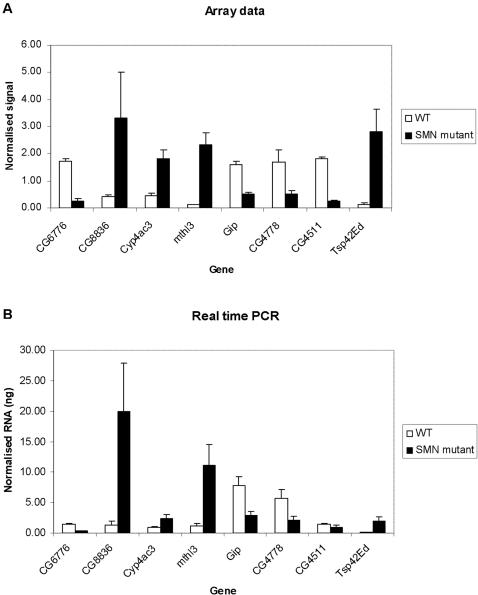
Bar graphs showing the gene expression changes across a set of eight genes comparing Affymetrix micro-array hybridisation (top) and Real-Time Polymerase Chain Reaction (bottom) results in whole 3rd instar larvae and muscle. Sample size was three for larvae and four for muscle. The eight genes were CG6776, CG8836, Cyp4ac3, mthl3, Gip, CG4778, CG4511 and Tsp42Ed. The changes measured this way showed that mRNA synthesis in whole 3rd instar SMN mutant larvae compared to WT larvae for CG6776 was decreased 3.57 fold (P<0.01), CG8836 was increased 14.6 fold (P<0.05), Cyp4ac3 was increased 2.7 fold (P<0.05), mthl3 was increased 8.9 fold (P<0.05). mRNA synthesis in SMN mutant larval muscle for Gip was decreased 2.7 fold (P<0.01), CG4778 was decreased 2.7 fold (<P0.01), CG4511 was decreased 1.6 fold (P<0.05), Tsp42Ed was increased 21.6 fold (P<0.05) . The RT-PCR analysis validates the micro-array data: the overall directions of change are all identical and the fold changes are within the same range

To assess the validity of our hybridisation results we examined the expression of a number of genes, randomly picked from our list, of differentially expressed genes using Real Time Polymerase Chain Reaction (RT-PCR) amplification. Strong correlation between PCR expression data and micro array values was found (Figure 3). All changes determined by RT-PCR were statistically significant in a Student's t-test (n = 3 and 4 for whole larvae and muscle respectively) and consistent with the direction of change reported by the micro-array analysis.

### Gene pathway changes

**Figure 4:**
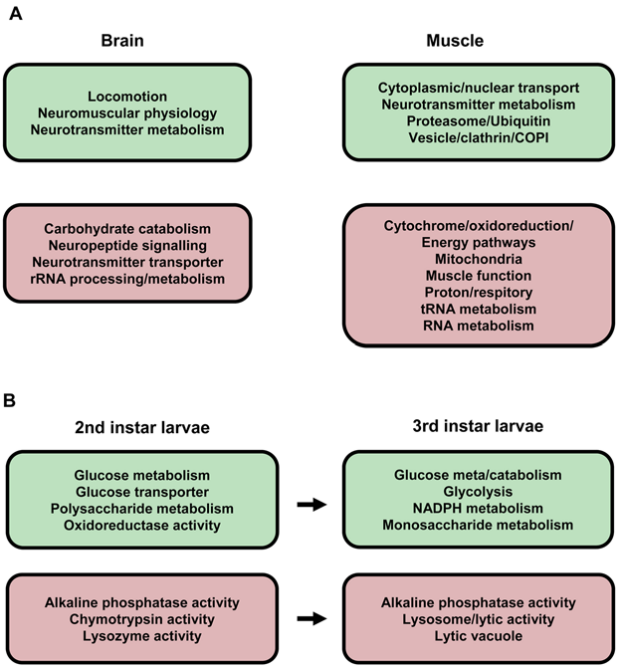
A) A selection of Gene Ontology terms associated with up (red)- or down (green) -regulated genes in muscle and brain tissues, comparing normal with *smn* mutant. B) A selection of Gene Ontology terms associated with up- or down-regulated genes in 2^nd^ and 3^rd^ instar larvae, comparing normal with *smn* mutant, same colour-coding. For both A) and B) full lists of these terms are shown in Supplemental Figure S3.

To explore functional changes within neuromuscular tissues we used Gene Ontology (GO) analysis to identify molecular pathways that were up- or down-regulated in *smn* mutant tissues versus control. Figure 4A shows a selection of categories in muscle and brain tissues where respectively ≥ 20% and ≥ 10% of genes per category are changed with a P value of ≤0.05 (Supplemental Figures S11 and S12 show the full list of GO terms in muscle and brain tissues, up and down regulated).

We find GO terms associated with NMJ physiology and locomotion down regulated in the brain tissue while terms involving general and rRNA metabolism are up regulated. In the muscle samples, terms associated with cellular transport and degradation are down-regulated. In addition neurotransmitter associated terms are down regulated.

Interestingly within the up-regulated genes in the muscle, very clear clustering of terms associated with energy metabolism is observed. Out of the highly significant terms (more than 50% of genes in a selected class are changing) more than half are associated with energy generation and mitochondrial activities. The other very clear grouping that is found up-regulated in the muscle is associated with RNA metabolism. RNA polymerase activity, RNA helicase as well as tRNA synthesis are found amongst the highly significant changes. The established function of SMN in RNA metabolism makes this an interesting observation. This possibly relates to the following. Comparing the muscle and brain samples, the gene expression changes in the muscle are often more significant and a considerable larger number of genes is found changed in muscle versus brain. These observations indicate that the effect of the reduction of SMN activity leads to a more pronounced response in muscle than in brain.

To study the temporal response to low SMN activity in the whole organism, we compared the GO terms in the 2^nd^ and 3^rd^ instar samples (using 20% cut off per category). The results are shown in Figure 4B (the full lists are shown in Supplemental Figures S13 and 14). Most changes in GO terms replicate across these samples, this strongly indicates that these reflect real responses to a reduction in SMN. In the down-regulated genes many terms fall into the category of metabolic, especially carbohydrate, enzyme activities. This indicates that the animals are possibly slowing their metabolic rate. Consistent with this we have observed that the mutant animals become emaciated.

In the up-regulated genes, interestingly, moving from the earlier time point to the later, terms associated with lytic activities are identified. This might indicate that the muscle tissues are very sensitive to the continued reduction in SMN activity and become degenerative (although no obvious signs are present on the tissue level).

### Genetic interactions

Our experiments show that expression of certain genes changes dramatically in *smn* depleted tissues. The function of SMN has been shown to involve the assembly of RNA-protein complexes [5], [12]. Rather than direct influences of SMN on gene transcription, the changes in gene expression we observe are therefore likely to be indirect due to the reduction of SMN activity. Such changes could be associated with an attempt within the tissues to maintain normal activity in the presence of depleted SMN function. Genes that are found up-regulated could perform a rescue function in the tissue. Reversely the down regulation of certain genes could be associated with the tissue reducing the activity of pathways that are disadvantageous at low levels of SMN. Agonists and antagonists to the function of these genes could aid the tissues in their struggle against phenotypes caused by low levels of SMN.

To further understand our findings and progress them into a functional realm we performed genetic experiments. We set out to probe if we could identify direct genetic interactions of loci corresponding to isolated genes, with *smn*. We combined an *smn* heterozygous mutant background with either mutant backgrounds (where available) and/or with over-expression constructs of random genes out of our lists. The selection of loci from our lists was random except for the fact that we were limited by the availability of genetic aberrations for selected genes. Table 3 shows the genes selected and the results of these genetic tests.

**Table 3:** Results of genetic experiments where *smn* mutant allele and alleles named in 2^nd^ column were combined in trans-heterozygous combination and welfare and survival were tested by counting the number of individuals of each genotype.

Gene/Down-regulated	Putative Disruption used	Result
l(2)k09913	BL12194	negative
CG5630	BL13026	negative
Zw	BL4153	negative
CG1941	BL10535	negative
CG17838	BL10721	negative
Pgd	BL11970	negative
l(1)G0320	BL11970	negative
CG1041	BL17954	negative
Bc	BL12276	negative
Lag1	BL12276	weak
CG3823	BL16489	negative
CG7115	BL14190	negative
Aldh-III	BL11342	very weak
CG12730	BL13004	negative
CG6028	BL14000	negative
**Gene/Up-regulated**		
GstE7	BL17923	negative
mthl3	BL5574	negative
CG12505	BL12770	negative
CPTI	BL13731	negative
CG6214	BL14544	negative
ab	BL10520	unclear
CG1882	BL13732	negative
Hmgs	BL11469	negative
stc	BL11408	very weak
kraken	BL13284	negative
CalpA	BL13868	negative
Rbp9	BL12836	negative
Hlc	BL4731	unclear
pelo	BL11757	negative
CG7461	BL18095	negative
CG11107	BL12911	unclear
und	BL6297	very weak
bowl	BL7094	weak
h	BL5529	negative
LK6	BL14934	negative

Results of genetic experiments where *smn* mutant allele and alleles named in 2^nd^ column were combined in trans-heterozygous combination and welfare and survival were tested by counting the number of individuals of each genotype. A positive score would be an outcome of more than 10% less individuals of the trans-heterozygous background compared to the normal alleles. Loci listed as negative did not show any genetic interaction in this test. The genes mentioned in column one are abbreviated and/or annotation or full names can be found on http://flybase.bio.indiana.edu/. On average more than 250 offspring were counted.

The test we used relies on subtle changes in the survival of the offspring. This test is relevant to the patient situation but it is only partially probing any functional interaction. On the other hand we examined a significant number of individuals in our test, generating a statistically more relevant outcome. We could also not assess if the aberrations we used here actually disrupt (or enhance) the activity of the indicated gene (note that numbered genes are only annotated as a gene and there are no functional data).

Despite these calculated set-backs, we clearly identified some genetic interactions with SMN in these tests and these will be discussed below.

## Discussion

Here we present a study of genome-wide transcription changes in a fly SMA model. We show that the technical difficulties in microarray experiments of working with small amounts of tissue material, inherent to working with Drosophila, are surmountable. This has led to statistically relevant and high quality data, which we independently verified using direct transcript level measurements.

By comparing the transcriptome from whole animal and individual tissues, samples isolated from virtually identical animals, we show that across the animal metabolic changes are taking place when SMN function is depleted. Across time, concomitant with the advent of SMA-like defects, certain metabolic genes are down-regulated. Interestingly, using Gene Ontology analysis, another set of genes that are associated with metabolic processes is identified in the muscle. These terms are mostly up-regulated and include many mitochondrion localized processes. It appears that the depletion of SMN function leads to metabolic changes in the animal.

Small Hsp genes provide activity to rescue cells under duress and three such genes are isolated in muscle and brain tissues as significantly changing in our experiments. Recently two such genes have been pinpointed as leading to a form of Charcot-Marie-Tooth disease, the most common inherited neuromuscular disease [13], [14]. These findings hint at a close and perhaps general interaction of small HSP genes with neuromuscular disorders.

We have previously shown [7] that the accumulation of neurotransmitter receptor at the SMN-depleted fly neuromuscular junction is disrupted. Here we show that this is a temporal process, while at the same time the levels of *smn* mRNA are slowly depleted. Our current experiments show that the transcription of a rate-limiting receptor subunit is increased, possibly reflecting an adjustment made in gene expression to increase receptor complex levels. In addition, our results also show for the first time, by using array results and direct measurements, that the levels of *smn* mRNA are higher in neuromuscular tissues, consistent with the higher sensitivity of these tissues to SMN depletion. Interestingly, comparing the reduction in GluR protein clusters with the reduction of subunit transcripts, it appears that the clustering problem is not based at the overall level of transcript. The suggested role of SMN in transcript localisation [15] thus indicates that SMN could act directly on transcripts associated with neurotransmitter function.

The Gene Ontology analysis allows the identification of functions affected in the system under study, rather than individual genes. It is clear the energy metabolism is isolated as affected in both types of analysis. In the muscle tissues metabolic processes associated with the mitochondrion are strongly affected. In amyotropic lateral sclerosis the case for an involvement of mitochondrial disturbances, a fatal neurodegenerative disorder, has been made extensively [16].

A second grouping of GO terms becomes obvious in the MAPP Finder results. These are mostly associated with neurotransmitter generation, levels and reception, as such involved in synaptic function. In brain tissue, many of the GO terms identified fall within this class, and they both increase as well as decrease in expression levels. In muscle however, the situation is skewed towards genes that are decreased in expression. These results point towards a function of SMN in the maintenance of synaptic function as driven by neurotransmitter generation, transfer and reception. As mentioned above, we have previously identified the loss of neurotransmitter receptor clusters as a highly consistent and relatively early defect in the *smn* mutant larvae. Clustering of receptors is at least partially driven by pre-synaptic activity [17] and maintenance of clustering has been shown to be dependent on glutamate (neurotransmitter at the fly neuromuscular junction) [18]. These results indicate that neurotransmitter metabolism might be a primary defect in the *smn* mutant and thus in SMA pathology.

Drosophila is a popular model system because of its traceable and rapid genetics. These qualities allow the discovery and characterisation of hitherto unknown functional relations between genes. We have used fly genetics to find new genes interacting with SMN, by selecting loci from our array results and combining these in a simple assay with the *smn* mutant locus in the fly. The number of genes (and available mutations) does not allow for an extensive study, however even in our limited analysis we have found evidence for genetic interaction with some genes.

Because of the large number of genes representing metabolic functions, our genetic analysis contains several of these. Interestingly however all loci that appear positive in our test (*Bc* (*Black cells*), Lag1, Aldh (Aldehyde dehydrogenase), *stc* (*shuttle craft*), *und* (*uninitiated*) and *bowl* (*brother of odd with entrails limited*) are in some way functionally associated with basic metabolism. Some are transcription oriented some are associated with basic enzymatic functions. We would conclude that although our test sample was small that metabolism is an important factor in determining the overall survival of the *smn* mutant animals and therefore possibly of SMA patients. On a more general level, we feel that this type of analysis, combining genomic technology with genetics will be useful in the functional analysis of (human disease) genes in simple model organisms. In addition, the continuous and rapid increase in molecularly characterised mutations in random genes in the fly, will make the resources reported here useful for further SMA functional studies.

## Materials and Methods

### Fly strain used

Our lab had obtained a mutant in the *smn* gene by analyzing candidate recessive-lethal mutants in the region where the gene is located based on genome annotation (73A7-9) [7]. Sequence analysis showed that one of the many lethal mutations in this region, the mutant l(3) 73Ao, contained a point mutation in the coding region of *smn*, resulting in a mis-sense mutation. Our lab recombined this *smn^73Ao^* mutant background with an isogenized stock to remove any unwanted lethal mutations in the genetic background (on the same chromosome). The resulting *smn^73Ao^* mutant was homozygous lethal at third larval stages.

The (normal) control used is the isogenic background on which the *smn* mutant background was recombined.

Both lines were maintained using standard conditions. For collection of material, flies were housed on apple juice plates with yeast in 25°C incubator with 10 hrs light-14 hrs dark cycle. Times larvae were isolated using genetic markers or Green Fluorescent Protein (expressed on the non-mutant allele, homozygous mutants will not light up green).

### Immunolabelling

For detection of specific proteins at the neuromuscular junction, we used antibodies against Smn protein (described in Thomas et al., under consideration) and syntaxin (see acknowledgements) on dissected tissues (as above) fixed using 4% paraformaldehyde. Incubations of antibodies (including secondary detection antibodies obtained from Molecular Probes/Invitrogen) were done in 1×PBS+0.1% Tween 20 and 0.3% saponin.

### Sample collection

In total we collected four samples for RNA isolation. The first sample is 2^nd^ instar larvae, second sample is 3^rd^ instar larvae. We selected female larvae (on basis of externally visible markers for 3^rd^ instar, for 2^nd^ instar we took 10 larvae total) in order to avoid any sex related gene expression changes between samples. The larvae were rinsed twice in 1xPBS (to wash off any bacteria or yeast) before RNA preparation (see below). The third and fourth samples are the 3^rd^ instar female larval brains and (abdominal ventral) muscle. Both tissues were dissected from the larvae in 1xPBS, and stored in RNAlater RNA Stabilization Reagent at −80°C. . Three biological replicates of larvae were prepared and four biological replicates of brain and muscle.

### RNA preparation

Total RNA was extracted from 2^nd^ and 3^rd^ instar larvae using the Qiagen RNeasy Mini Kit. The animals were homogenized in 350 µL RLT buffer (+ß-Mercapto-Ethanol: ME) with a hand held homogenizer (5 mm probe blades) and the manufacturers protocol was followed for further isolation.

Total RNA was extracted from brain and muscle tissues using the Qiagen RNeasy Micro Kit, as above.

RNA concentration and integrity were assessed by absorbance at 260 and 280 nm and by an Agilent Bioanalyzer RNA 6000 Nano Assay (data not shown).

### Sample labelling and hybridisation to Affymetrix array

Samples were prepared for Affymetrix analysis from whole 2^nd^ and 3^rd^ instar larvae using 1 ug starting RNA. The Affymetrix One-Cycle Target Labelling and Control kit (P/N900493) was used according to protocol 701027 Rev. 4 in the Affymetrix Eukaryotic Sample and Array Processing Manual. For Drosophila larval brain and muscle 100 ng and 40 ng starting RNA were used respectively. To successfully generate sufficient labelled cRNA for microarray analysis two rounds of amplification were necessary. For this the Affymetrix Two-Cycle Target Labelling and Control kit was used (P/N 900494) according to the manufacturer's instructions described in the Affymetrix Eukaryotic Sample and Array Processing Manual version 701027 Rev.5. For the first cycle of *in vitro* transcription the Ambion MEGAscript T7 kit (P/N 1334) was used as detailed in the Affymetrix manual. Labelled cRNA was hybridised to Drosophila 1 chips and arrays were washed and then scanned using the Affymetrix GeneChip Scanner3000.

### Analysis of differential gene expression

Microarray data were GCRMA normalised independently for each tissue using GeneSpring GX (Agilent). Differentially expressed genes were identified using a Welch t test with a p value cut off of ≤0.05 and a fold change difference between wild type and mutant *smn* of ≥1.5.

### Real-Time RT-PCR (SYBR green)

Total RNA from WT and *smn* mutant whole 3^rd^ instar larvae and muscle was used as a template for RT-PCR to independently assess the validity of Affymetrix GeneChip data. 10 ng 3^rd^ instar total larval RNA and 1 ng larval muscle RNA per sample was used to synthesise first-strand cDNA, using random hexamers and SuperScript III reverse transcriptase (Invitrogen). The cDNA was used for quantitative real-time polymerase chain reaction (PCR) amplification with Sybr Green chemistry (Applied Biosystems). Primer Express software (Applied Biosystems) was used to design primers from the RefSeq sequences. The target gene names, RefSeq numbers and primer sequences are shown in Table 4. Triplicate cDNA template samples were amplified and analyzed in the Prism 7000 sequence detection system (Applied Biosystems). The thermal cycle conditions were 50°C for 2 min, 95°C for 10 min, 40 cycles of 15 s at 95°C followed by 1 min at 60°C. Immediately after the amplification, melt curve protocols were performed to ensure that primer-dimers and other non-specific products had been minimized or eliminated. A standard curve of cycle thresholds using serial dilutions of cDNA samples was established and used to calculate the relative abundance of the target gene in WT and mutant *smn* samples. Values were normalized to gamma-Tubulin (at map position 23C), we used this gene as its expression was not changing in our Affymetrix data. Minus RT samples tested simultaneously with experimental samples by quantitative RT-PCR with Sybr Green consistently yielded no amplification below 30 cycles using the above protocol.

**Table 4:** Probe and Primer Sequences for Real-Time PCR

mRNA		Sequence (5′-3′)	Accession number
Tubulin 23C	Forward	AGTGCGGCAATCAAATTGG	NM_057456
	Reverse	GCTCCAGGCACAATCTTTTCC	
Mthl3	Forward	GAAAGTGAAGAAGGAAGCACAAAAC	NM_145335
	Reverse	GGACAAGCCCATGATGATAAAGA	
CG6776	Forward	CGGGCACAGGACAAGATTCT	NM_139977
	Reverse	TGCCCTGCACAAGGATGTT	
CG4511	Forward	AGAAGCAGGAGTGGCTCAGAAA	NM_141769
	Reverse	AAACAATGTTGGGCGACTTTTT	
Tsp42Ed	Forward	GCGGCGTTTTTGTGAAATATG	NM_078906
	Reverse	TGATGGAGCCGAAGGTGATAA	
CG8836	Forward	AGCGGGCTCCTATTCCTACAA	NM_136929
	Reverse	CTGCTGCTGGTTTCCAATGTT	
Cyp4ac3	Forward	GCCATGCTGGACACTCTTTTG	NM_135074
	Reverse	GGTATCGTATCCCCCGAACAT	
CG4778	Forward	CCCTACAACATTGACTGCATGAA	NM_135495
	Reverse	GGCTTCTCGTGGCCAAAGT	
Gip	Forward	CGATAGCCTTAGAGCCAGTAAAATG	NM078551
	Reverse	ATTCCAGCGGCTTTGGAGTAT	

### Real-time RT-PCR (Probe)

Real-time quantitative PCR was performed by using TaqMan TAMRA probe chemistry on the ABI Prism 7000 Sequence Detector System (Applied Biosystems). Primers and probes were designed using Primer Express V2.0 software. Thermal Cycling parameters were 95°C for 10 min, followed by 40 cycles of 95°C for 1 minute, 59°C for 1 minute, and 72°C for 45 s. Aliquots of 50 µl consisting of primers, probes, cDNA and TaqMAN RT PCR master mix were run in triplicate on the Applied Biosystems 7000 sequence detection system. Primers were used at 900 nM concentration and probes at 20 nM concentration for both target and control. A serial dilution for each target gene was run on each plate in triplicate. The dilution series consisted of cDNA at 2 ng 1 ng, 0.5 ng 0.2 ng, 0.1 ng, 0.05 ng, and 0.02 ng concentrations. Data was analysed using the comparative relative quantification method and samples were normalised to both Actin42A and GAPDH. Three plates were run for this experiment.

## Supporting Information

Figure S1 cRNA yields and quality from decreasing amounts of starting RNA. A) The yield of cRNA falls steeply when the amount of starting material goes below 1 ng. B) The size distribution is smaller when the 2 cycle labelling kit is used for 100 ng RNA compared with cRNA transcripts generated using the 1 cycle kit for 5 ug starting RNA. The smaller the amount of starting RNA the smaller the transcripts that are generated. C) Scatter plots show some genes decrease in signal when smaller amounts starting amounts of RNA are used. This was shown to be due to 5′ truncation of cRNA transcripts, preventing complete hybridisation to the rarer 5′ probe sets (data not shown). However the loss of gene expression signal resulting from reduced starting material may not always translate into changed gene expression changes.
(0.70 MB DOC)

Figure S2 Genes identified in brain tissue, not in 2nd and 3rd instar larvae, corresponding to 1st and 5th rows in Table 1a.
(0.09 MB XLS)

Figure S3 Genes identified in 2nd instar larvae and brain tissue, not in 3rd instar larvae, corresponding to 2nd and 6th rows in Table 1a.
(0.02 MB XLS)

Figure S4 Genes identified in 3rd instar larvae and brain, not in 2nd instar larvae, corresponding to 3rd and 7th rows Table 1a.
(0.02 MB XLS)

Figure S5 Genes identified in brain, 2nd and 3rd instar larvae, corresponding to 4th and 8th rows in Table 1a
(0.02 MB XLS)

Figure S6 Genes identified in muscle tissue, not in 2nd and 3rd instar larvae, corresponding to 1st and 5th rows in Table 1b.
(0.26 MB XLS)

Figure S7 Genes identified in 2nd instar larvae and muscle tissue, not in 3rd instar larvae, corresponding to 2nd and 6th rows in Table 1b.
(0.02 MB XLS)

Figure S8 Genes identified in 3rd instar larvae and muscle, not in 2nd instar larvae, corresponding to 3rd and 7th rows in Table 1b.
(0.03 MB XLS)

Figure S9 Genes identified in muscle, 2nd and 3rd instar larvae, corresponding to 4th and 8th rows in Table 1b
(0.02 MB XLS)

Figure S10 Genes identified as in common between brain and muscle samples, corresponding to last two rows in Table 1c.
(0.06 MB XLS)

Figure S11 Gene Ontology (GO) collective terms for genes that are differentially regulated in SMN mutant muscle with a 1.5 fold cut-off. Terms are selected only if 20% or more of genes in a GO term are changed.
(0.08 MB XLS)

Figure S12 Gene Ontology (GO) collective terms for genes that are differentially regulated in mutant SMN brain with a 1.5 fold cut-off. GO terms are selected only if 10% or more of genes in a GO term are changed.
(0.03 MB PPT)

Figure S13 Gene Ontology (GO) collective terms for genes that are differentially regulated in SMN mutant 2nd instar larvae with a 1.5 fold cut-off. Terms are selected only if 20% or more of genes in a GO term are changed
(0.02 MB XLS)

Figure S14 Gene Ontology (GO) collective terms for genes that are differentially regulated in SMN mutant 3rd instar larvae with a 1.5 fold cut-off. Terms are selected only if 20% or more of genes in a GO term are changed
(0.02 MB XLS)
