# Correction: Genome-Wide Expression Analysis of a Spinal Muscular Atrophy Model: Towards Discovery of New Drug Targets

**DOI:** 10.1371/annotation/f45a5dc0-e317-4886-8c5b-1569c79a5f08

**Published:** 2008-04-22

**Authors:** Sheena Lee, Arzu Sayin, Ruben J. Cauchi, Stuart Grice, Howard Burdett, Dilair Baban, Marcel van den Heuvel

There is an error in the authorship of this paper. Ruben J. Cauchi should be included as an author and is affiliated with institution 1. He should appear between AS and SG in the author list.

