# Supplementary material for: Genome-Wide Expression Analysis of a Spinal Muscular Atrophy Model: Towards Discovery of New Drug Targets
Source: PLoS One. 2008 Jan 2;3(1):e1404. doi: 10.1371/journal.pone.0001404 (PMC2151137; doi:10.1371/journal.pone.0001404)
Supplement: Figure S1 — cRNA yields and quality from decreasing amounts of starting RNA. A) The yield of cRNA falls steeply when the amount of starting material goes below 1 ng. B) The size distribution is smaller when the 2 cycle labelling kit is used for 100 ng RNA compared with cRNA transcripts generated using the 1 cycle kit for 5 ug starting RNA. The smaller the amount of starting RNA the smaller the transcripts that are generated. C) Scatter plots show some genes decrease in signal when smaller amounts starting amounts of RNA are used. This was shown to be due to 5′ truncation of cRNA transcripts, preventing complete hybridisation to the rarer 5′ probe sets (data not shown). However the loss of gene expression signal resulting from reduced starting material may not always translate into changed gene expression changes. (0.70 MB DOC) [file pone.0001404.s001.doc]

**Figure S1**

A)

|  | **Starting RNA** | **cRNA yield (ug)** |
| --- | --- | --- |
| **Affymetrix 1-cycle** | **5ug** | **121** |
| **Affymetrix 2-cycle** | **100ng** | **143** |
| **Affymetrix 2-cycle** | **10ng** | **113** |
| **Affymetrix 2-cycle** | **1ng** | **97.8** |
| **Affymetrix 2-cycle** | **0.1ng** | **20.4** |
| **Affymetrix 2-cycle** | **0.01ng** | **2.0** |
|  |  |  |

**B)**

**C)**
